# Evaluation of Smartphone Technology on Spatiotemporal Gait in Older and Diseased Adult Populations

**DOI:** 10.3390/s24175839

**Published:** 2024-09-09

**Authors:** Coby Contreras, Ethan C. Stanley, Chanc Deschamps-Prescott, Susan Burnap, Madison Hopkins, Bennett Browning, Jesse C. Christensen

**Affiliations:** Veterans Affairs Salt Lake City Health Care System, Department of Physical Therapy and Athletic Training, University of Utah, Salt Lake City, UT 84108, USA

**Keywords:** validity, reliability, agreement, smartphone application, motion analysis system, gait

## Abstract

*Objective:* Advancements in smartphone technology provide availability to evaluate movement in a more practical and feasible manner, improving clinicians’ ability to diagnose and treat adults at risk for mobility loss. The purpose of this study was to evaluate the validity and reliability of a smartphone application to measure spatiotemporal outcomes during level (primary) and uphill/downhill (secondary) walking with and without an assistive device for older adults (OAs), Parkinson’s Disease (PD) and cerebrovascular accident (CVA) populations. *Methods:* A total of 50 adults (OA = 20; PD = 15; CVA = 15) underwent gait analysis at self-selected gait speeds under 0-degree, 5-degree uphill and 5-degree downhill environments. The validity and reliability of the smartphone outcomes were compared to a motion-capture laboratory. Bland–Altman analysis was used to evaluate limits of agreement between the two systems. Intraclass correlation coefficients (ICCs) were used to determine absolute agreement, and Pearson correlation coefficients (r) were used to assess the strength of the association between the two systems. *Results:* For level walking, Bland–Altman analysis revealed relatively equal estimations of spatiotemporal outcomes between systems for OAs without an assistive device and slight to mild under- and overestimations of outcomes between systems for PD and CVA with and without an assistive device. Moderate to very high correlations between systems (without an assistive device: OA r-range, 0.72–0.99; PD r-range, 0.87–0.97; CVA r-range, 0.56–0.99; with an assistive device: PD r-range, 0.35–0.98; CVA r-range, 0.50–0.99) were also observed. Poor to excellent ICCs for reliability between systems (without an assistive device: OA ICC range, 0.71–0.99; PD ICC range, 0.73–0.97; CVA ICC range, 0.56–0.99; with an assistive device: PD ICC range, 0.22–0.98; CVA ICC range, 0.44–0.99) were observed across all outcomes. *Conclusions:* This smartphone application can be clinically useful in detecting most spatiotemporal outcomes in various walking environments for older and diseased adults at risk for mobility loss.

## 1. Introduction

Gait impairments significantly affect our ability to perform activities of daily living and maintain independence, particularly in older adults (OAs) and adults with neurological diseases such as Parkinson’s Disease (PD) and those who have sustained a cerebrovascular accident (CVA) [1,2,3]. Pronounced gait impairments are commonly observed in OAs and adults with PD and CVA [4,5], making these populations high risk for future disability and falls. Gait impairments during level walking are strongly predictive of fall-related disability, and it is the most common task humans engage in during activities of daily living [6]. However, when adults engage in activities of daily living, they encounter various terrains, such as uphill and downhill walking. Uphill and downhill walking accounts for greater fall risk and is more metabolically demanding compared to level walking [7,8]. These varying walking terrains are a contributor to why over one-third of adults over 65 years sustain a fall annually, resulting in an estimated 9 million fall-related injuries each year [9]. Alarmingly, an estimated USD 50 billion is spent per year on fall-related medical costs in OAs and adults with neurological disease, with fatal fall-related costs consuming USD 754 million of the total costs [10].

The gold standard for evaluating gait impairments is an optical-motion-capture laboratory equipped with high-speed cameras and instrumented force plates [11]. However, these methods are not routinely available for most clinicians and patients, making their utility for evaluating gait impairments impractical. Alternatively, clinicians can resort to visual observation, portable force plates and inertial measurement units to measure gait impairments. However, these methods can be relatively expensive, unreliable and require a skilled operator for data interpretation [12,13].

Advancements in inertial sensor technology have led to embedded accelerometers and gyroscopes in common smartphones [14]. Using smartphone technology for gait impairment evaluation could be a cost-effective, portable and practical alternative to a motion-capture laboratory, while also allowing for evaluation in a patient’s own natural environment [15]. Smartphone applications that evaluate gait impairments are available [16,17,18,19,20,21]. However, few smartphone applications have been rigorously tested and compared to the motion-capture laboratory. Additionally, less research has been conducted on advanced spatiotemporal outcomes in both OAs and adults with neurological disease during level walking, and no study has compared measurement error during uphill and downhill walking.

Understanding the utility of smartphone applications could serve as a possible indicator for future fall risk and subsequent disability, particularly in high-risk populations. It is also essential to evaluate gait impairment within walking conditions (i.e., level, uphill and downhill) that OAs and adults with neurological disease encounter daily. This information could further allow for remote monitoring of high-risk adults and provide clinicians with technology to diagnose and treat gait impairments that can be translatable to any hospital, outpatient and/or home setting.

Therefore, the primary purpose of this study was to evaluate the validity and reliability of a smartphone application (OneStep, Tel Aviv, Israel) to measure spatiotemporal outcomes during level walking with and without use of an assistive device in OA, PD and CVA populations. The secondary purpose of this study was to evaluate the validity and reliability of the spatiotemporal outcomes during uphill and downhill walking with and without use of an assistive device in OA, PD and CVA populations. The gold standard for validation was the 3D optical-motion-capture laboratory (Vicon, Oxford Metrics Ltd., Oxford, UK). Our hypothesis was that the smartphone application would provide accurate estimates of the participant’s gait pattern that could be used in clinical applications and long-term remote-monitoring studies. This study aimed to (1) determine measurement error between innovative smartphone technology and the gold-standard motion-capture laboratory in a relatively robust sample of aging and neurodegenerative adults, (2) evaluate various sloped walking environments that are commonly observed in activities of daily living that require greater metabolic and mechanical demand to the lower limbs relative to traditional level walking and (3) provide the first sensitive measurement study of spatiotemporal outcomes in mobility-impaired adults with use of an assistive device in various sloped walking environments.

## 2. Methods

### 2.1. Participants

This study employed a cross-sectional design with a cohort of OA, PD and CVA participants. The eligibility requirements for the OA cohort were adults ≥65 years of age with no history of any health conditions that would significantly affect their walking or balance abilities. The eligibility requirements for the PD and CVA cohorts were adults ≥18 years of age, diagnosed with PD or CVA, able to ambulate ≥50 feet without an assistive device and with use of a single-point cane; adults that needed more assistance than a single-point cane for independent walking were excluded. All participants were recruited from the University of Utah Healthcare System and Center of Aging registry (Salt Lake City, UT, USA), and procedures were approved by the University of Utah Institutional Review Board (IRB#00160259). All eligible participants provided written, informed consent prior to participating in the study. The precision approach was used for sample size determination [22,23,24], and assuming intraclass correlation coefficients (ICCs) = 0.80, the sample size (n = 20) and number of raters (single rater) provide a 95% confidence interval around ICCs of width 0.37 (ICC ± 0.18). These data seemed to be within acceptable precision for our purposes, so the sample size and number of raters were adequate.

### 2.2. Experimental Procedures

**Figure 1:**
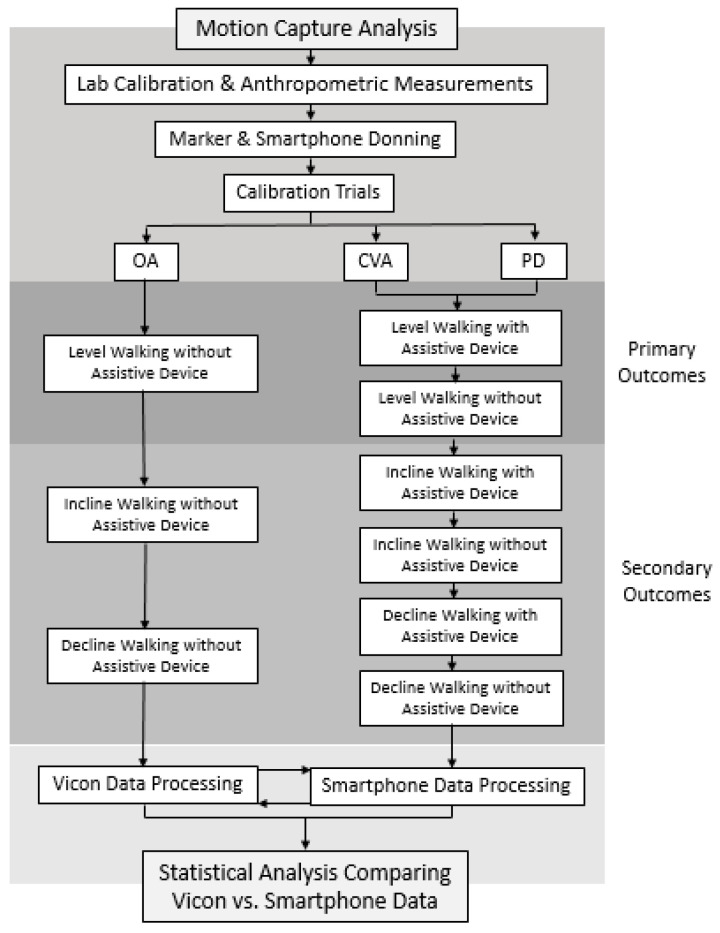
Flow chart for data collection and processing.

**Figure 2:**
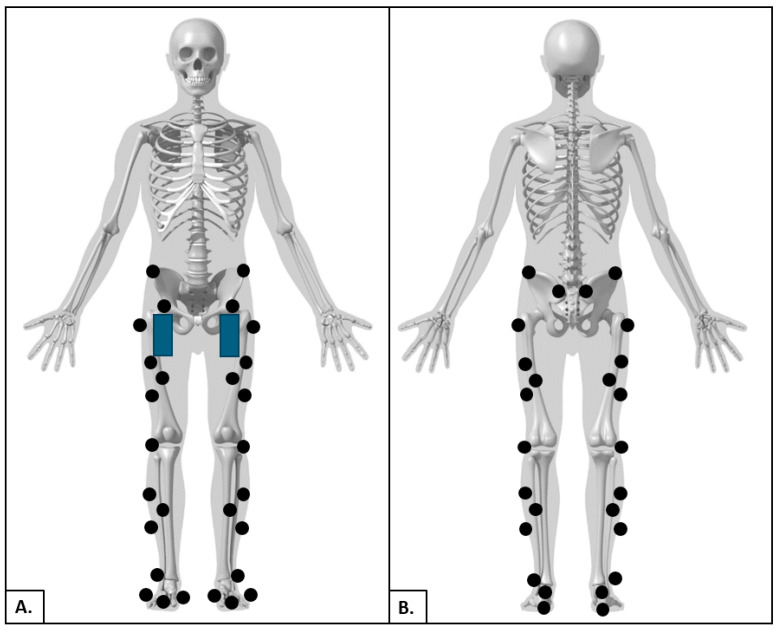
Marker and smartphone placement for modified Plug-In-Gait marker set and iPhone anterior thigh placement ((**A**) anterior, (**B**) posterior). Image supplied by C-Motion, Inc. (Germantown, MD, USA) used with permission.

All participants completed gait analysis at the Motion Capture Core Facility at the University of Utah, Department of Physical Therapy and Athletic Training. Data collection was performed in a single session lasting no more than two hours. The experimental protocol comprised four sequential steps as described below (Figure 1):

1. *System Initialization*: Gait analysis was performed using a 10-camera motion analysis system sampling at 200 Hz (Vicon Motion Systems; Oxford, UK). Kinetic data were obtained using a dual-belt instrumented treadmill (Bertec; Columbus, OH, USA) with sampling at 1000 Hz. Spatiotemporal data were recorded and synchronized using Nexus v2.15.0 software (Vicon, Oxford Metrics Ltd., Oxford, UK). The treadmill was equipped with a harness system to ensure the safety of each participant during the walking trials. Two lateral handrails that were not instrumented were also installed for further safety support. The motion-capture cameras and the instrumented treadmill were initialized as recommended by the manufacturer [25]. The initialization protocol consisted of calibration of the cameras within the capture volume, leveling the treadmill, setting the volume origin and zeroing the force plates of the treadmill [25].
2. *Participant Preparation*: Each participant was fitted with compressive clothing, and retroreflective markers were affixed to bony landmarks throughout the pelvis and bilateral lower limbs, including the iliac crests, anterior/posterior superior iliac spine, greater trochanters, lateral femoral condyles, lateral malleoli, head of the 5th metatarsals and upper and lower aspects of the heels (Figure 2). Two non-rigid clusters with 4 non-collinear markers were placed at the lateral side of each thigh and shank segment. A single research physical therapist affixed all markers to each participant in the study. This was implemented to reduce the risk of marker placement error across the cohort. The modified Plug-In-Gait marker set (Vicon, Oxford Metrics Ltd., Oxford, UK) defined 1 HAT segment (combined head, arms and trunk), 1 pelvis segment, 2 thigh segments, 2 shank segments and 2 foot segments. Marker locations were used for attributing coordinate systems for each segment and were positioned as previously described [15]. Two smartphones (iPhone SE, Apple Inc., Cupertino, CA, USA) were also affixed to the right and left anterolateral thighs of each participant. The OneStep smartphone application was downloaded and activated on each device prior to formal data collection. The OneStep smartphone application uses data collected by the smartphone’s built-in sensors to measure gait parameters. Acceleration and angular data were collected by the smartphone’s sensors at a sampling rate of 100 Hz, and those data were analyzed by proprietary algorithms to measure the spatiotemporal variables for each stride. An upper-body harness was securely fastened to the participants. The participants were then directed onto the treadmill, and the harness was connected to an overhead support system.
3. *Calibration Trials:* Two calibration trials were collected with each participant on the treadmill. *Static Calibration*: The participants were asked to stand stationary for five seconds with legs shoulder-width apart and arms out in front with elbows slightly bent [26]. Static calibration markers were removed after static calibration was completed. *Joint Center Calibration*: The participants were asked to swing each leg, individually, in a clock pattern from twelve to six or six to twelve, depending on the side. Then, the participants were asked to flex and extend each knee, individually, to the full available range of motion 5 times. Lastly, the participants were asked to circumduct each ankle, individually, within the full available range of motion 5 times [26].
4. *Walking Trials*: The participants walked at a self-selected pace on the instrumented treadmill within three environments: (1) 0-degree level slope, (2) 5-degree uphill slope and (3) 5-degree downhill slope. The self-selected level gait speed was acquired using the 5 m walk test [27]. Participants were instructed to walk at a normal speed over the course of a standard 10 m measured walkway and two marked areas 5 m apart. Three separate trials of the 5 m walk test were conducted and their average was used for the self-selected level gait speed trials during formal data collection. The 5-degree slope angle was selected to replicate common adults’ experience during activities of daily living and is an environment that could be safely tested, while requiring greater mechanical demand to the lower limbs compared to level walking [28]. All participants completed a 1–2 min warm-up period during each gait environment before formal testing. Once participants confirmed they felt comfortable with the gait environment, they were instructed to walk as naturally as possible as data was collected. Participants walked continuously at a self-selected speed for 2–3 min. Participants were then provided a 5 to 10 min rest period prior to beginning the next gait environment to minimize the risk of fatigue. The OA participants performed all gait environments without a single-point cane. The PD and CVA participants performed all gait environments with and without a single-point cane. Rating of perceived exertion and numeric pain rating scale scores were recorded following the completion of each session. Trials in which participants lost their balance were excluded. For each walking environment, participants were brought up to the desired self-selected gait speed and were provided 30 s to acclimate before formal data collection was conducted. The first 30 successful steps on each limb, following the acclimate period, were averaged and used for statistical analysis.

### 2.3. Data Processing

*Motion-capture system processing*: Marker trajectories and ground reaction force data were synchronized, recorded and pre-processed using Vicon Nexus software v2.15.0 (Vicon, Oxford Metrics Ltd., Oxford, UK). Marker trajectory data were collected at 200 Hz, and ground reaction force data were collected at 1000 Hz. Pre-processing involved labeling the markers, defining segments and calculating segment dimensions using the static and functional calibration trials. Once the markers were labeled, gaps in the marker trajectories were filled using rigid-body, spline and pattern-fill algorithms [25]. Gait events such as heel strike and toe off were detected and marked using ground reaction force signals [25]. A low-pass Butterworth filter with a cut-off frequency of 6 Hz was applied for the marker trajectories, and 20 Hz was applied for the analog force plate data. A residual analysis was used to determine the cut-off frequency for the low-pass Butterworth filter [29]. Low-pass filtering was applied to the marker trajectories and analog force plate data before further analysis was performed. Customized Python scripts were used to acquire spatiotemporal outcomes (see *Spatiotemporal Outcomes* section for details) and were compared to the smartphone application spatiotemporal outcomes. Two independent researchers blinded to the extent of the study identified all Vicon gait events and spatiotemporal outcomes. *Smartphone application processing*: Post-processing and extraction of spatiotemporal variables were conducted using OneStep software v4.1 (Celloscope Ltd., Tel Aviv, Israel). A senior researcher processed and exported all smartphone application gait events and spatiotemporal outcomes.

### 2.4. Spatiotemporal Outcomes

Spatiotemporal outcomes collected during self-selected gait trials were based on past studies [30]. These outcomes were defined as the following:

- Double-limb stance (%): The time that both feet are in contact with the ground simultaneously, summed as the time elapsed during two periods of double-limb support in the gait cycle and calculated as a percentage of the gait cycle.
- Single-limb stance (%): The time only one foot was in contact with the ground, summed as the time elapsed between initial contact and toe off on the same foot and calculated as a percentage of the gait cycle.
- Swing (%): The time only one foot was off the ground, summed as the time elapsed between the last contact of the current toe off to initial contact of the next heel strike of the same foot and calculated as a percentage of the gait cycle.
- Step length (m): Anterior–posterior distance from the heel of one footprint to the heel of the opposite footprint.
- Stride length (m): Anterior–posterior distance between heels of two consecutive footprints of the same foot (left to left, right to right); two steps (e.g., a right step followed by a left step) comprise one stride or one gait cycle.
- Cadence (steps/min): Number of steps per minute, sometimes referred to as step rate.
- Gait speed (m/s): Calculated by dividing the distance walked by the ambulation time.

### 2.5. Statistical Analysis 

Descriptive statistics were used to determine demographic characteristics of the participants in this study. The Bland–Altman method was used to visualize the reproducibility and determine the limits of agreement (LoA) between the smartphone application and motion analysis system for each spatiotemporal outcome within each gait environment (validity) [31]. Plots of mean ± SD 1.96 were used to demonstrate the 95% confidence interval (CI) of agreement (limit of agreement) between the two systems [32]. The intraclass correlation coefficients (ICCs) were used to determine absolute agreement between the two systems (reliability). The ICC accounts for both the differences between systems and the degree of correlation [33]. The Pearson correlation coefficients (r) were used to assess the strength of the association between the two systems.

Interpreting the strength of the reliability according to ICCs was as follows: poor reliability (ICCs < 0.50), moderate reliability (ICCs = 0.50–0.75), good reliability (ICCs = 0.75–0.90) and excellent reliability (ICCs > 0.90) [34]. Interpreting the strength of the association according to Pearson correlations was as follows: negligible (r = 0.0–0.3), low (r = 0.3–0.5), moderate (r = 0.5–0.7), high (r = 0.7–0.9) and very high (r = 0.9–1.0) [35]. Analyses were performed using STATA v17.0 statistical software package (College Station, TX, USA).

## 3. Results

### 3.1. Participants

A total of 95 adults were screened for enrollment, of which 32 declined and 13 were excluded due to having a history of significant health issues affecting walking and balance ability (8 reported significant health comorbidities; 5 were wheelchair bound). In the end, 50 adults (OA = 20, PD = 15, CVA = 15) were enrolled (Table 1).

### 3.2. Validity

**Figure 3:**
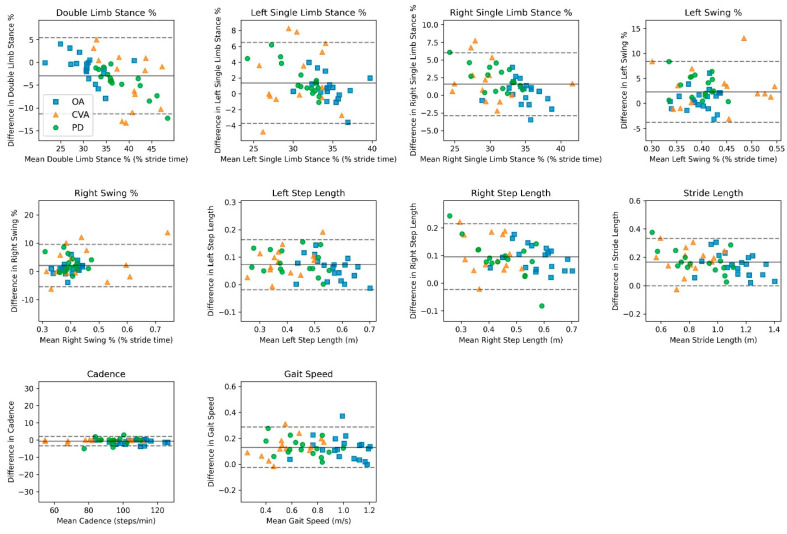
Bland–Altman plots comparing the smartphone application and motion-capture system measurements in assessing spatiotemporal outcomes for level walking without an assistive device across OAs, PD and CVA. Mean bias is displayed as a solid line, and 95% limits of agreement are displayed as dashed lines.

For the OA level walking without an assistive device data, agreement showed relatively equal estimations for the time-based outcomes and a slight overestimation of the smartphone application compared to the motion analysis system on the distance-based outcomes (Table 2). For *time-based outcomes* (double-limb stance %, single-limb stance %, swing %), the mean bias range was −1.03 to 0.61% [LoA range: 3.39 to 5.80]. For *distance-based outcomes* (right/left step length, stride length), the mean bias range was 0.06 to 0.15 m (LoA range: 0.09 to 0.16). The mean bias for cadence was −0.97 steps/minute (LoA: 2.42), and the mean bias for gait speed was 0.12 m/second (LoA: 0.19). Pearson correlations between the smartphone application and motion analysis system across all spatiotemporal outcomes ranged from high to very high (range, r = 0.72–0.99 Table 2). Bland–Altman analyses revealed agreement between the smartphone application and motion analysis system, as displayed in Figure 3.

For the adults with PD level walking without an assistive device data, agreement showed a slight overestimation of the smartphone application compared to the motion analysis system for most spatiotemporal outcomes (Table 2). For *temporal-based outcomes* (double-limb stance %, single-limb stance %, swing %), the mean bias range was −4.23 to 2.53% (LoA range: 3.62 to 6.32). For *spatial-based outcomes* (right/left step length, stride length), the mean bias range was 0.07 to 0.18 m (LoA range: 0.09 to 0.17). The mean bias for cadence was −0.42 steps/minute (LoA: 4.10), and the mean bias for gait speed was 0.14 m/second (LoA: 0.13). Pearson correlations between the smartphone application and motion analysis system across all spatiotemporal variables ranged from high to very high (range, r = 0.87–0.97 Table 2). Bland–Altman analyses revealed agreement between the smartphone application and motion analysis system, as displayed in Figure 3.

**Figure 4:**
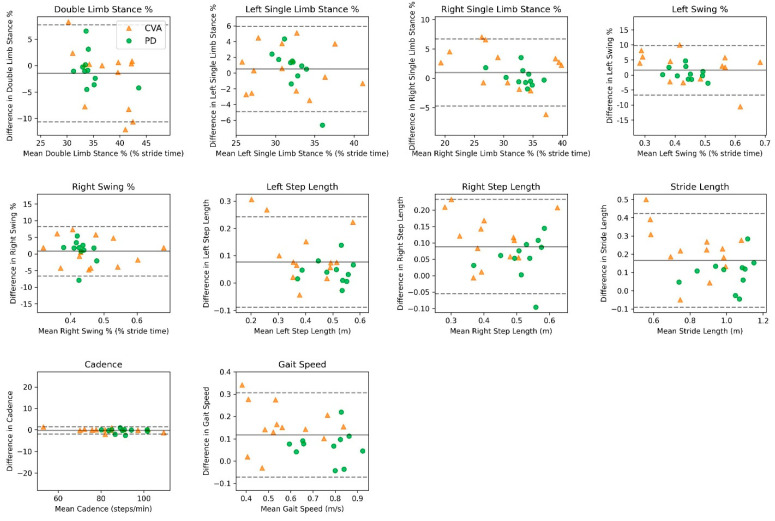
Bland–Altman plots comparing the smartphone application and motion-capture system measurements in assessing spatiotemporal outcomes for level walking with an assistive device across PD and CVD. Mean bias is displayed as a solid line, and 95% limits of agreement are displayed as dashed lines.

For the adults with PD level walking with an assistive device data, agreement showed a slight overestimation of the smartphone application compared to the motion analysis system for most spatiotemporal outcomes (Table 3). For *temporal-based outcomes* (double-limb stance %, single-limb stance %, swing %), the mean bias range was −0.79 to 0.44% (LoA range: 3.06 to 6.83). For *spatial-based outcomes* (right/left step length, stride length), the mean bias range was 0.04 to 0.11 m (LoA range: 0.08 to 0.16). The mean bias for cadence was −0.42 steps/minute (LoA: 2.08), and the mean bias for gait speed was 0.09 m/second (LoA: 0.14). Pearson correlations between the smartphone application and motion analysis system across all spatiotemporal variables ranged from low to very high (range, r = 0.35–0.98, Table 3). Bland–Altman analyses revealed agreement between the smartphone application and motion analysis system, as displayed in Figure 4.

For adults with CVA level walking without an assistive device data, agreement showed slight to mild under and overestimations of the smartphone application compared to the motion analysis system across the spatiotemporal outcomes (Table 2). For *temporal-based outcomes* (double-limb stance %, single-limb stance %, swing %), the mean bias range was −0.78 to 0.64% (LoA range: 3.36 to 6.96). For *spatial-based outcomes* (right/left step length, stride length), the mean bias ranged from 0.06 to 0.17 m (LoA range: 0.08 to 0.14). The mean bias for cadence was −0.13 steps/minute (LoA: 1.89), and the mean bias for gait speed was 0.13 m/second (LoA: 0.15). Pearson correlations between the smartphone application and motion analysis system across all spatiotemporal variables ranged from moderate to very high (range, r = 0.56–0.99 Table 2). Bland–Altman analyses revealed agreement between the smartphone application and motion analysis system, as displayed in Figure 3.

For the adults with CVA level walking with an assistive device data, agreement showed slight overestimations of the smartphone application compared to the motion analysis system across most of the spatiotemporal outcomes (Table 3). For *temporal-based outcomes* (double-limb stance %, single-limb stance %, swing %), the mean bias range was −1.97 to 1.47% (LoA range: 5.71 to 11.2). For *spatial-based outcomes* (right/left step length, stride length), the mean bias range was 0.10 to 0.20 m (LoA range: 0.18 to 0.31). The mean bias for cadence was −0.09 steps/minute (LoA: 1.56), and the mean bias for gait speed was 0.16 m/second (LoA: 0.23). Pearson correlations between the smartphone application and motion analysis system across all spatiotemporal variables ranged from moderate to very high (range, r = 0.50–0.99 Table 3). Bland–Altman analyses revealed agreement between the smartphone application and motion analysis system, as displayed in Figure 4.

Supplemental Tables S1–S4 and Figures S1–S4 provide full validity details for the uphill and downhill walking trials with and without an assistive device across all cohorts.

### 3.3. Reliability

For the OA level walking without an assistive device data, good to excellent inter-system reliability was observed (range, ICC = 0.71–0.99; Table 2). For the adults with PD level walking without an assistive device data, good to excellent inter-system reliability was observed (range, ICC = 0.73–0.97; Table 2). For adults with CVA level walking without an assistive device data, moderate to excellent inter-system reliability was observed (range, ICC = 0.56–0.99; Table 2).

For adults with PD level walking with an assistive device data, poor to excellent inter-system reliability was observed (range, ICC = 0.22–0.98; Table 2). For adults with CVA level walking with an assistive device data, moderate to excellent inter-system reliability was observed (range, ICC = 0.44–0.99; Table 2).

Supplemental Tables S1–S4 and Figures S1–S4 provide full reliability details for the incline and decline walking trials with and without an assistive device across all cohorts.

## 4. Discussion

The primary purpose of this study was to evaluate the validity and reliability of a smartphone application to measure spatiotemporal outcomes during level walking with and without use of an assistive device in OA, PD and CVA populations. The secondary purpose of this study was to evaluate the validity and reliability of the spatiotemporal outcomes during uphill and downhill walking with and without use of an assistive device in OA, PD and CVA populations. This study found the smartphone application provided good to excellent agreement with the majority of the spatiotemporal outcomes during level walking with and without use of an assistive device for the OA, PD and CVA groups. Additionally, the level of reliability across outcomes varied depending on the group and environment. Generally, the spatiotemporal outcomes showed better reliability in level walking without use of an assistive device in the OA group compared to both level walking with and without an assistive device in the PD and CVA groups. High-reliability measures were observed in cadence and gait speed among nearly all groups and environments. Lower-reliability measures were observed particularly in the temporal-based outcomes expressed as a percentage of the gait cycle in the OA and CVA groups. To our knowledge, this is the first study to examine validity and reliability of a smartphone application compared to the gold-standard motion-capture laboratory targeting different walking environments with and without use of an assistive device in both older and diseased adult populations.

Excellent agreement for cadence during level walking without use of an assistive device was observed across all groups. The results showed low measurement error for the temporal-based outcomes in the OA and CVA groups, but less agreement was observed in the PD group. The variance in agreement for double-limb stance percentage increased the most in the PD group. However, no systematic magnitude errors were observed across other phase-based outcomes based on Bland–Altman analysis. The results also showed relatively low measurement error for the spatial-based measures and gait speed outcomes across all groups, which is consistent with prior studies [36,37,38]. Relatively good to excellent reliability was also observed during level walking without use of an assistive device in the OA and PD groups. However, reduced reliability was observed in the temporal-based outcomes, particularly in the CVA group walking without use of an assistive device.

Low measurement error was also observed in most spatiotemporal outcomes for level walking with use of an assistive device in the PD and CVA groups. However, reduced agreement was observed in stride length and gait speed outcomes, particularly in the CVA group. Adults with CVA commonly present with asymmetric gait due to select impairments (e.g., sensory deficits, reduced motor control function, plantar-flexor spasticity) [39,40], which can increase dependency on the nonparetic limb [41]. Additionally, walking with the use of an assistive device on a treadmill at a constant speed is an unnatural environment for most adults with CVA and could have accounted for some of the observed variability, despite adequate warm-up and safety measures. Moderate to excellent reliability was observed for the spatial-based, cadence and gait speed outcomes in both the PD and CVA groups. However, mixed findings were observed in temporal-based outcomes, possibly due to variability in measurement error or individual gait pattern changes over the trial. The lower reliability, particularly in relatively phase-based outcomes (i.e., single-limb stance and swing %), is in line with previous work [42,43]; however, our findings did show encouragingly better overall agreement compared to other studies that conclude poor agreement in temporal-based outcomes [42,43,44,45,46].

Relatively good agreement for single-limb percentage, swing percentage, step length and cadence outcomes during uphill walking with and without use of an assistive device was observed across all groups. The highest errors occurred in double-limb stance percentage, stride length and gait speed outcomes, particularly in the PD and CVA groups. Lower measurement error was observed in the temporal-based outcomes in the OA group compared to the PD and CVA groups. Better agreement was observed for the temporal-based outcomes during incline walking with use of an assistive device compared to without an assistive device in the PD group. This is likely due to the slower gait speed and additional support provided during walking with the use of an assistive device. Additionally, temporal-based outcomes in the CVA group also showed better agreement in the uphill walking with use of an assistive device compared to without an assistive device, despite no change in gait speed between trials. Intuitively, this makes sense as more support results in less variability within gait pattern characteristics and better agreement in measuring the targeted outcome.

Relatively good agreement for single-limb percentage, swing percentage, step length, cadence and gait speed outcomes during downhill walking with and without the use of an assistive device was observed across all groups. Similar to uphill walking, the highest errors occurred in double-limb stance percentage and stride length outcomes, particularly in the PD and CVA groups. However, better agreement was observed in downhill walking compared to uphill walking. This could be partially explained by the increased metabolic and physical demand required for uphill walking, compared to downhill walking, particularly in the mobility-impaired groups. This likely resulted in less variability in gait pattern characteristics during downhill walking. Similar to uphill walking, lower measurement error was observed in the temporal-based outcomes in the OA group compared to the PD and CVA groups. Better agreement was also observed for the temporal-based outcomes during downhill walking with use of an assistive device compared to without an assistive device in both the PD and CVA groups.

To date, little is known about the validity and reliability of detecting spatiotemporal outcomes in various sloped walking environments across mobility-impaired adult populations. The data obtained from this study fill gaps in the existing literature by (1) determining measurement error between innovative smartphone technology and the gold standard motion-capture laboratory in a relatively robust sample of aging and neurodegenerative adults, (2) evaluating various sloped walking environments that are commonly observed in activities of daily living that require greater metabolic and mechanical demand to the lower limbs relative to traditional level walking and (3) providing the first sensitive measurement study of spatiotemporal outcomes in mobility-impaired adults with use of an assistive device in various sloped walking environments. Alternatively, the findings of this study must be evaluated considering some limitations and specific methodological choices: (1) the motion-capture laboratory allowed for complete control over the study protocol and acquisition of gait outcomes. However, this constrained laboratory environment could influence gait outcomes [47] and lower the external validity of the results obtained in this study, as accuracy of gait outcomes in different environments might be affected in more natural settings due to a higher variability of activities. (2) We only collected data with unobstructed straight walking in the laboratory. No turnings or other natural movements were evaluated, which should be considered in future studies in order to assess the accuracy in less constrained movement environments. (3) While our cohort was adequately powered to answer our primary purpose, increased sample size with different demographics (i.e., body mass, morbidities, etc.) could increase the rigor of these methods. (4) We used an instrumented treadmill for evaluation of gait outcomes between systems, which could affect the participants’ gait patterns [48]. The smartphone application and algorithms were independent of overground and treadmill walking, but this could have some influence and should be evaluated in future study.

## 5. Conclusions

These data suggest that the OneStep smartphone application holds promise in providing valid and reliable spatiotemporal outcomes across different walking environments within both older and diseased adult populations. Clinicians can cautiously use the smartphone application as a more practical tool for detecting gait impairments in mobility-impaired populations compared to the traditional motion-capture laboratory.

## Figures and Tables

**Table 1:** Descriptive characteristics.

Variable	OA (n = 20)	PD (n = 15)	CVA (n = 15)
Age, y	74.2 (4.2)	72.6 (9.1)	52.3 (26.7)
Sex, % male	60	60	87
Weight, kg	75.9 (16.5)	84.1 (11.7)	91.5 (17.5)
Height, m	1.71 (0.13)	1.71 (0.14)	1.77 (0.08)
BMI, kg/m^2^	25.8 (4.4)	28.9 (4.8)	28.9 (4.8)
PROMIS Global Health—Physical	55.6 (4.5)	42.0 (7.1)	44.2 (8.2)
PROMIS Global Health—Mental	56.3 (6.3)	48.7 (9.3)	49.6 (10.8)
UCLA Activity Scale	6 (3–9)	4.8 (2–7)	4.2 (1–8)
CCI	3.5 (1.1)	2.9 (1.0)	3.5 (1.5)
Hoehn and Yahr	--	2.5 (0.7)	--

Note: Values represented as mean (SD), unless otherwise stated. Values for UCLA activity scale represented as mean (range). BMI, body mass index; PROMIS, Patient-Reported Outcomes Measurement Information System; UCLA, University of California Los Angeles; CCI, Charlson Comorbidity Index.

**Table 2:** Spatiotemporal comparison between the motion-capture system and smartphone application for level walking without an assistive device.

Population/Variable	Vicon Mean (SD)	OneStep Mean (SD)	Mean Bias (95% CI)	LoA (95% CI)	*r* ^ꞙ^	ICC ^¥^ (95% CI)
**Older Adults**						
Double-Limb Stance, %	31.3	30.3	−1.03	±5.80	0.80	0.77
	(4.90)	(3.65)	(−2.39, 0.32)	(3.44, 8.15)		(0.51, 0.90)
Left Single-Limb Stance, %	34.1	34.7	0.60	±3.41	0.72	0.71
	(2.47)	(2.01)	(−0.20, 1.39)	(2.03, 4.80)		(0.40, 0.87)
Right Single-Limb Stance, %	34.5	34.9	0.43	±3.45	0.73	0.71
	(2.51)	(2.01)	(−0.38, 1.23)	(2.05, 4.85)		(0.40, 0.87)
Left Swing, %	34.5	34.9	0.41	±3.48	0.72	0.71
	(2.52)	(2.01)	(−0.40, 1.22)	(2.07, 4.89)		(0.40, 0.87)
Right Swing, %	34.1	34.7	0.61	±3.39	0.72	0.71
	(2.45)	(2.01)	(−0.18, 1.40)	(2.02, 4.76)		(0.40, 0.87)
Left Step Length, m	0.53	0.58	0.06	±0.09	0.85	0.83
	(0.08)	(0.06)	(0.04, 0.08)	(0.05, 0.12)		(0.63, 0.93)
Right Step Length, m	0.52	0.61	0.09	±0.09	0.86	0.85
	(0.08)	(0.07)	(0.07, 0.11)	(0.05, 0.12)		(0.66, 0.93)
Stride Length, m	1.05	1.2	0.15	±0.16	0.88	0.86
	(0.16)	(0.13)	(0.11, 0.18)	(0.09, 0.22)		(0.69, 0.94)
Cadence, steps/min	105.8	104.8	−0.97	±2.42	0.99	0.99
	(10.7)	(10.6)	(−1.54, −0.41)	(1.43, 3.40)		(0.99, 0.99)
Gait Speed, m/s	0.93	1.05	0.12	±0.19	0.87	0.86
	(0.19)	(0.16)	(0.07, 0.16)	(0.11, 0.26)		(0.69, 0.94)
**Parkinson’s Disease**						
Double-Limb Stance, %	40.5	36.2	−4.23	±6.32	0.95	0.81
	(6.4)	(3.7)	(−5.98, −2.47)	(3.29, 9.35)		(0.54, 0.93)
Left Single-Limb Stance, %	30.1	31.8	1.69	±4.20	0.90	0.73
	(3.6)	(1.8)	(0.54, 2.86)	(2.19, 6.21)		(0.38, 0.90)
Right Single-Limb Stance, %	29.3	31.9	2.53	±3.63	0.87	0.81
	(3.4)	(2.4)	(1.52, 3.53)	(1.89, 5.37)		(0.53, 0.93)
Left Swing, %	29.3	31.9	2.52	±3.62	0.87	0.81
	(3.5)	(2.4)	(1.51, 3.52)	(1.88, 5.36)		(0.54, 0.93)
Right Swing, %	30.1	31.8	1.71	±4.18	0.90	0.74
	(3.6)	(1.8)	(0.55, 2.86)	(2.18, 6.19)		(0.38, 0.90)
Left Step Length, m	0.38	0.45	0.07	±0.09	0.91	0.91
	(0.10)	(0.09)	(0.05, 0.10)	(0.05, 0.13)		(0.75, 0.96)
Right Step Length, m	0.38	0.48	0.10	±0.11	0.89	0.84
	(0.11)	(0.07)	(0.07, 0.13)	(0.06, 0.16)		(0.59, 0.94)
Stride Length, m	0.77	0.94	0.18	±0.17	0.93	0.90
	(0.21)	(0.16)	(0.13, 0.22)	(0.09, 0.25)		(0.74, 0.96)
Cadence, steps/min	94.0	93.6	−0.42	±4.10	0.97	0.97
	(8.7)	(9.3)	(−1.55, 0.71)	(2.14, 6.07)		(0.92, 0.99)
Gait Speed, m/s	0.60	0.74	0.14	±0.13	0.94	0.93
	(0.18)	(0.16)	(0.10, 0.17)	(0.07, 0.19)		(0.82, 0.97)
**Cerebrovascular Accident**						
Double-Limb Stance, %	30.4	30.5	0.15	±6.96	0.71	0.68
	(4.96)	(3.64)	(−1.47, 1.78)	(4.14, 9.78)		(0.35, 0.85)
Left Single-Limb Stance, %	34.6	33.8	−0.78	±3.36	0.72	0.67
	(2.43)	(1.66)	(−1.57, 0.01)	(2.00, 4.73)		(0.34, 0.85)
Right Single-Limb Stance, %	34.9	35.5	0.64	±5.10	0.57	0.56
	(2.68)	(2.81)	(−0.55, 1.83)	(3.04, 7.17)		(0.18, 0.80)
Left Swing, %	34.9	35.5	0.63	±5.11	0.56	0.56
	(2.68)	(2.81)	(−0.57, 1.82)	(3.04, 7.18)		(0.17, 0.80)
Right Swing, %	34.6	33.8	−0.77	±3.36	0.72	0.67
	(2.43)	(1.66)	(−1.56, 0.01)	(1.99, 4.72)		(0.33, 0.85)
Left Step Length, m	0.48	0.55	0.06	±0.08	0.92	0.89
	(0.09)	(0.07)	(0.05, 0.08)	(0.05, 0.11)		(0.76, 0.95)
Right Step Length, m	0.47	0.57	0.10	±0.09	0.88	0.88
	(0.09)	(0.09)	(0.08, 0.12)	(0.05, 0.13)		(0.72, 0.95)
Stride Length, m	0.96	1.12	0.17	±0.14	0.92	0.92
	(0.18)	(0.16)	(0.13, 0.20)	(0.08, 0.20)		(0.81, 0.96)
Cadence, steps/min	105.54	105.42	−0.13	±1.89	0.99	0.99
	(11.5)	(11.5)	(−0.57, 0.32)	(1.13, 2.67)		(0.99, 0.99)
Gait Speed, m/s	0.86	0.98	0.13	±0.15	0.92	0.91
	(0.17)	(0.16)	(0.09, 0.16)	(0.09, 0.21)		(0.78, 0.96)

Note: SD, standard deviation; ICC, intraclass correlation coefficient; CI, confidence interval; LoA, limits of agreement; r, Pearson correlation coefficient. A positive mean bias value indicates the smartphone application (OneStep) overestimated the variable compared to the motion-capture system (Vicon). A negative mean bias value indicates the smartphone application underestimated the variable compared to the motion-capture system. ^ꞙ^ < 0.30, (negligible); 0.30–0.50, (low); 0.50–0.70, (moderate); 0.70–0.90, (high); >0.90, (very high). ^¥^ < 0.50, (poor reliability); 0.50–0.75, (moderate reliability); 0.75–0.90, (good reliability); >0.90, (excellent reliability).

**Table 3:** Spatiotemporal comparison between the motion-capture system and smartphone application for level walking with an assistive device.

Population/Variable	Vicon Mean (SD)	OneStep Mean (SD)	Mean Bias (95% CI)	LoA (95% CI)	*r* ^ꞙ^	ICC ^¥^ (95% CI)
**Parkinson’s Disease**						
Double-Limb Stance, %	35.1	34.3	−0.79	±6.83	0.60	0.57
	(4.2)	(3.0)	(−3.24, 1.65)	(2.67, 11.07)		(−0.04, 0.87)
Left Single-Limb Stance, %	32.1	32.5	0.44	±5.85	0.35	0.22
	(3.1)	(1.1)	(−1.65, 2.52)	(2.23, 9.48)		(−0.43, 0.72)
Right Single-Limb Stance, %	32.7	33.1	0.36	±3.06	0.86	0.84
	(3.0)	(2.4)	(−0.74, 1.45)	(1.16, 4.95)		(0.50, 0.96)
Left Swing, %	32.7	33.1	0.36	±3.06	0.86	0.84
	(3.0)	(2.4)	(−0.73, 1.46)	(1.15, 4.96)		(0.50, 0.96)
Right Swing, %	32.1	32.5	0.43	±5.83	0.35	0.22
	(3.1)	(1.1)	(−1.65, 2.51)	(2.22, 9.45)		(−0.43, 0.72)
Left Step Length, m	0.47	0.51	0.04	±0.09	0.81	0.81
	(0.07)	(0.07)	(0.01, 0.07)	(0.04, 0.15)		(0.41, 0.95)
Right Step Length, m	0.47	0.54	0.07	±0.08	0.88	0.82
	(0.05)	(0.07)	(0.04, 0.10)	(0.03, 0.13)		(0.44, 0.95)
Stride Length, m	0.95	1.06	0.11	±0.16	0.84	0.83
	(0.12)	(0.15)	(0.05, 0.17)	(0.06, 0.26)		(0.46, 0.95)
Cadence, steps/min	90.1	89.7	−0.42	±2.08	0.98	0.98
	(7.0)	(7.1)	(−1.16, 0.33)	(0.79, 3.37)		(0.95, 0.99)
Gait Speed, m/s	0.71	0.80	0.09	±0.14	0.83	0.83
	(0.12)	(0.12)	(0.03, 0.13)	(0.05, 0.23)		(0.47, 0.95)
**Cerebrovascular Accident**						
Double-Limb Stance, %	38.1	36.1	−1.97	±11.2	0.50	0.44
	(6.4)	(3.8)	(−5.22, 1.27)	(5.69, 16.85)		(0.08, 0.78)
Left Single-Limb Stance, %	31.3	31.8	0.56	±5.73	0.82	0.82
	(4.8)	(4.8)	(−1.09, 2.21)	(2.86, 8.60)		(0.53, 0.93)
Right Single-Limb Stance, %	30.5	31.9	1.40	±7.37	0.87	0.85
	(7.6)	(6.1)	(−0.71, 3.53)	(3.74, 11.05)		(0.61, 0.95)
Left Swing, %	30.4	31.9	1.47	±7.25	0.88	0.86
	(7.7)	(6.1)	(−0.62, 3.56)	(3.67, 10.88)		(0.63, 0.95)
Right Swing, %	31.3	31.8	0.56	±5.71	0.82	0.82
	(4.8)	(4.8)	(−1.09, 2.20)	(2.86, 8.57)		(0.54, 0.94)
Left Step Length, m	0.35	0.45	0.10	±0.20	0.68	0.64
	(0.13)	(0.09)	(0.05, 0.16)	(0.10, 0.30)		(0.20, 0.87)
Right Step Length, m	0.37	0.47	0.10	±0.18	0.67	0.65
	(0.12)	(0.09)	(0.05, 0.15)	(0.09, 0.28)		(0.20, 0.87)
Stride Length, m	0.73	0.93	0.20	±0.31	0.74	0.69
	(0.23)	(0.15)	(0.11, 0.29)	(0.16, 0.46)		(0.28, 0.89)
Cadence, steps/min	84	83.9	−0.09	±1.56	0.99	0.99
	(14.5)	(14.1)	(−0.55, 0.36)	(0.77, 2.34)		(0.99, 0.99)
Gait Speed, m/s	0.49	0.65	0.16	±0.23	0.78	0.76
	(0.18)	(0.15)	(0.09, 0.22)	(0.12, 0.35)		(0.41, 0.91)

Note: SD, standard deviation; ICC, intraclass correlation coefficient; CI, confidence interval; LoA, limits of agreement; r, Pearson correlation coefficient. A positive mean bias value indicates the smartphone application (OneStep) overestimated the variable compared to the motion-capture system (Vicon). A negative mean bias value indicates the smartphone application underestimated the variable compared to the motion-capture system. ^ꞙ^ < 0.30, (negligible); 0.30–0.50, (low); 0.50–0.70, (moderate); 0.70–0.90, (high); >0.90, (very high). ^¥^ < 0.50, (poor reliability); 0.50–0.75, (moderate reliability); 0.75–0.90, (good reliability); >0.90, (excellent reliability).

## Data Availability

The data presented in this study are available on request from the corresponding author due to institutional HIPAA regulations.

## Supplementary Materials

The following supporting information can be downloaded at: https://www.mdpi.com/article/10.3390/s24175839/s1, Table S1. Spatiotemporal comparison between motion capture system and smartphone application for uphill walking without an assistive device. Table S2. Spatiotemporal comparison between motion capture system and smartphone application for uphill walking with an assistive device. Table S3. Spatiotemporal comparison between motion capture system and smartphone application for downhill walking without an assistive device. Table S4. Spatiotemporal comparison between motion capture system and smartphone application for downhill walking with an assistive device. Figure S1. Bland-Altman plots comparing the smartphone application and motion capture system measurements in assessing spatiotemporal outcomes for incline walking without an assistive device across OA, PD, CVA. Mean bias is displayed as a solid line and 95% limits of agreement are displayed as dashed lines. Figure S2. Bland-Altman plots comparing the smartphone application and motion capture system measurements in assessing spatiotemporal outcomes for incline walking with assistive device across PD and CVD. Mean bias is displayed as a solid line and 95% limits of agreement are displayed as dashed lines. Figure S3. Bland-Altman plots comparing the smartphone application and motion capture system measurements in assessing spatiotemporal outcomes for decline walking without an assistive device across OA, PD, CVA. Mean bias is displayed as a solid line and 95% limits of agreement are displayed as dashed lines. Figure S4. Bland-Altman plots comparing the smartphone application and motion capture system measurements in assessing spatiotemporal outcomes for decline walking with assistive device across PD and CVD. Mean bias is displayed as a solid line and 95% limits of agreement are displayed as dashed lines.

## Abbreviations

Older adults, OAs; Parkinson’s Disease, PD; cerebrovascular accident, CVA; limits of agreement, LoA; intraclass correlation coefficients, ICCs; Pearson correlations, r.
