# Supplementary material for: Evaluation of Smartphone Technology on Spatiotemporal Gait in Older and Diseased Adult Populations
Source: Sensors (Basel). 2024 Sep 9;24(17):5839. doi: 10.3390/s24175839 (PMC11397937; doi:10.3390/s24175839)
Supplement: Supplementary file 1 [file sensors-24-05839-s001.zip › sensors-3128624-supplementary.pdf]

Table S1. Spatiotemporal comparison between motion capture system and smartphone application for uphill walking without an assistive device.

Population/Variable	Vicon Mean (SD)	OneStep Mean (SD)	Mean Bias (95% CI)	LoA (95% CI)	r^f	ICC[¥] (95% CI)
**Older Adults**						
Double Limb Stance, %	33.9 (4.8)	31.2 (3.6)	-2.68 (-4.50, -0.86)	±7.78 (4.62, 10.90)	0.61	0.58 (0.20, 0.81)
Left Single Limb Stance, %	32.9 (2.4)	34.1 (1.6)	1.09 (-0.02, 2.20)	±4.74 (2.82, 6.66)	0.37	0.34 (-0.10, 0.67)
Right Single Limb Stance, %	33.1 (2.6)	34.6 (2.2)	1.54 (0.66, 2.43)	±3.81 (2.27, 5.35)	0.70	0.69 (0.37, 0.86)
Left Swing, %	33.1 (2.6)	34.6 (2.2)	1.54 (0.64, 2.44)	±3.84 (2.28, 5.40)	0.69	0.69 (0.36, 0.86)
Right Swing, %	32.9 (2.4)	34.1 (1.6)	1.09 (-0.01, 2.20)	±4.72 (2.81, 6.63)	0.37	0.35 (-0.09, 0.68)
Left Step Length, m	0.52 (0.08)	0.60 (0.07)	0.09 (0.06, 0.11)	±0.10 (0.06, 0.14)	0.81	0.79 (0.54, 0.91)
Right Step Length, m	0.51 (0.08)	0.65 (0.06)	0.14 (0.12, 0.16)	±0.09 (0.06, 0.13)	0.85	0.82 (0.61, 0.92)
Stride Length, m	1.03 (0.17)	1.26 (0.13)	0.22 (0.18, 0.26)	±0.18 (0.10, 0.25)	0.87	0.84 (0.64, 0.93)
Cadence, steps/min	103.43 (11.3)	102.96 (11.2)	-0.47 (-0.75, 0.18)	±1.20 (0.72, 1.69)	0.99	0.99 (0.99, 0.99)
Gait Speed, m/s	0.89 (0.18)	1.08 (0.17)	0.19 (0.15, 0.24)	±0.17 (0.10, 0.24)	0.89	0.88 (0.73, 0.95)
**Parkinson's Disease**						
Double Limb Stance, %	42.2 (7.0)	36.0 (4.5)	-6.24 (-8.44, -4.04)	±7.95 (4.14, 11.76)	0.85	0.77 (0.45, 0.91)
Left Single Limb Stance, %	28.8	32.1	3.24	±4.83	0.78	0.73

	(3.8)	(2.6)	(1.90, 4.58)	(2.60, 7.14)		(0.37, 0.90)
Right Single Limb Stance, %	28.8<br>(3.5)	31.8<br>(2.4)	2.98<br>(1.85, 4.11)	±4.09<br>(2.13, 6.05)	0.83	0.77<br>(0.45, 0.91)
Left Swing, %	28.8<br>(3.5)	31.8<br>(2.4)	2.98<br>(1.85, 4.12)	±4.11<br>(2.13, 6.08)	0.83	0.77<br>(0.45, 0.91)
Right Swing, %	28.8<br>(3.8)	32.1<br>(2.6)	3.24<br>(1.91, 4.58)	±4.82<br>(2.58, 7.13)	0.78	0.73<br>(0.37, 0.90)
Left Step Length, m	0.39<br>(0.13)	0.46<br>(0.1)	0.07<br>(0.05, 0.10)	±0.11<br>(0.06, 0.16)	0.93	0.90<br>(0.72, 0.96)
Right Step Length, m	0.38<br>(0.12)	0.52<br>(0.08)	0.14<br>(0.10, 0.18)	±0.14<br>(0.07, 0.20)	0.87	0.79<br>(0.49, 0.92)
Stride Length, m	0.77<br>(0.25)	0.99<br>(0.18)	0.22<br>(0.15, 0.28)	±0.22<br>(0.12, 0.33)	0.93	0.87<br>(0.66, 0.95)
Cadence, steps/min	90.6<br>(8.1)	90.9<br>(7.9)	0.33<br>(-0.59, 1.25)	±3.33<br>(1.72, 4.93)	0.97	0.97<br>(0.93, 0.99)
Gait Speed, m/s	0.58<br>(0.21)	0.75<br>(0.16)	0.17<br>(0.13, 0.22)	±0.17<br>(0.08, 0.25)	0.93	0.90<br>(0.74, 0.96)
**Cerebrovascular Accident**						
Double Limb Stance, %	44.7<br>(6.8)	38.2<br>(5.1)	-6.54<br>(-10.2, -2.79)	±12.98<br>(6.52, 19.47)	0.44	0.42<br>(-0.11, 0.77)
Left Single Limb Stance, %	27.5<br>(3.7)	30.8<br>(2.7)	3.3<br>(0.98, 5.63)	±8.00<br>(3.9, 11.97)	0.27	0.26<br>(-0.29, 0.68)
Right Single Limb Stance, %	27.6<br>(4.8)	30.8<br>(3.8)	3.23<br>(1.45, 5.00)	±6.14<br>(3.11, 9.21)	0.77	0.75<br>(0.39, 0.91)
Left Swing, %	27.6<br>(4.8)	30.8<br>(3.8)	3.23<br>(1.44, 5.01)	±6.16<br>(3.15, 9.24)	0.78	0.75<br>(0.39, 0.91)
Right Swing, %	27.5<br>(3.8)	30.8<br>(2.8)	3.30<br>(0.98, 5.63)	±8.00<br>(4.00, 11.97)	0.27	0.26<br>(-0.29, 0.68)

Left Step Length, m	0.31 (0.07)	0.45 (0.07)	0.14 (0.11, 0.18)	±0.11 (0.06, 0.17)	0.74	0.74 (0.37, 0.90)
Right Step Length, m	0.35 (0.11)	0.50 (0.07)	0.15 (0.11, 0.19)	±0.15 (0.07, 0.22)	0.77	0.70 (0.30, 0.89)
Stride Length, m	0.66 (0.16)	0.96 (0.13)	0.29 (0.25, 0.35)	±0.17 (0.08, 0.26)	0.84	0.83 (0.56, 0.94)
Cadence, steps/min	83.2 (19.4)	83.1 (19.1)	-0.15 (-1.12, 0.83)	±3.39 (1.69, 5.09)	0.99	0.99 (0.98, 0.99)
Gait Speed, m/s	0.47 (0.16)	0.67 (0.17)	0.19 (0.14, 0.25)	±0.199 (0.09, 0.29)	0.83	0.83 (0.55, 0.94)

Note: Standard deviation (SD); Intraclass correlation coefficient (ICC); confidence interval (CI); limits of agreement (LoA); Pearson correlation coefficient (r). A positive mean bias value indicates the smartphone application (OneStep) overestimated the variable compared to the motion capture system (Vicon). A negative mean bias value indicates the smartphone application underestimated the variable compared to the motion capture system.

[f]<0.30 (negligible); 0.30-0.50 (low); 0.50-0.70 (moderate); 0.70-0.90 (high); >0.90 (very high)

[¥]<0.50 (poor reliability); 0.50-0.75 (moderate reliability); 0.75-0.90 (good reliability); >0.90 (excellent reliability)

Table S2. Spatiotemporal comparison between motion capture system and smartphone application for uphill walking with an assistive device.

Population/Variable	Vicon Mean (SD)	OneStep Mean (SD)	Mean Bias (95% CI)	LoA (95% CI)	r^f	ICC[¥] (95% CI)
**Parkinson's Disease**						
Double Limb Stance, %	33.8 (6.2)	34.4 (3.3)	0.59 (-4.09, 5.28)	±13.10 (5.00, 21.22)	0.17	0.14 (-0.49, 0.68)
Left Single Limb Stance, %	32.9 (3.5)	32.9 (1.8)	0.03 (-2.71, 2.77)	±7.67 (2.97, 12.43)	0.07	0.05 (-0.56, 0.63)
Right Single Limb Stance, %	33.2 (4.3)	32.5 (2.4)	-0.66 (-3.18, 1.86)	±7.06 (2.76, 11.44)	0.57	0.49 (-0.14, 0.84)
Left Swing, %	33.2 (4.3)	32.5 (2.4)	-0.65 (-3.17, 1.88)	±7.07 (2.69, 11.45)	0.57	0.49 (-0.14, 0.84)
Right Swing, %	32.9 (3.5)	32.9 (1.8)	0.013 (-2.72, 2.75)	±7.667 (2.95, 12.42)	0.07	0.06 (-0.56, 0.63)
Left Step Length, m	0.50 (0.08)	0.54 (0.07)	0.04 (-0.00, 0.08)	±0.12 (0.04, 0.19)	0.76	0.73 (0.23, 0.92)
Right Step Length, m	0.47 (0.07)	0.58 (0.06)	0.12 (0.07, 0.14)	±0.09 (0.04, 0.16)	0.77	0.76 (0.30, 0.93)
Stride Length, m	0.98 (0.15)	1.12 (0.13)	0.15 (0.08, 0.21)	±0.19 (0.07, 0.31)	0.78	0.77 (0.32, 0.93)
Cadence, steps/min	86.3 (6.9)	86.4 (7.2)	0.05 (-0.46, 0.56)	±1.42 (0.54, 2.29)	0.99	0.99 (0.97, 0.99)
Gait Speed, m/s	0.70 (0.14)	0.81 (0.11)	0.11 (0.06, 0.16)	±0.15 (0.06, 0.24)	0.86	0.84 (0.49, 0.95)
**Cerebrovascular Accident**						
Double Limb Stance, %	41.7 (7.7)	36.0 (4.3)	-5.72 (-10.40, -0.96)	±16.42 (8.12, 24.60)	0.16	0.14 (-0.40, 0.60)
Left Single Limb Stance, %	29.8	32.5	2.73	±9.37	0.53	0.52

	(5.2)	(4.3)	(0.03, 5.43)	(4.77, 14.06)		(0.01, 0.81)
Right Single Limb Stance, %	28.3 (7.7)	31.3 (5.3)	2.95 (-0.17, 6.07)	±10.75 (5.35, 16.09)	0.71	0.66 (0.23, 0.87)
Left Swing, %	28.3 (7.6)	31.3 (5.3)	2.97 (-0.13, 6.08)	±10.73 (5.33, 16.08)	0.71	0.67 (0.23, 0.88)
Right Swing, %	29.8 (5.2)	32.5 (4.3)	2.71 (-0.00, 5.42)	±9.39 (4.69, 14.08)	0.53	0.52 (0.01, 0.81)
Left Step Length, m	0.31 (0.14)	0.47 (0.09)	0.16 (0.11, 0.21)	±0.17 (0.09, 0.26)	0.81	0.75 (0.39, 0.91)
Right Step Length, m	0.34 (0.11)	0.52 (0.09)	0.18 (0.13, 0.22)	±0.16 (0.08, 0.24)	0.71	0.70 (0.30, 0.89)
Stride Length, m	0.66 (0.22)	1.00 (0.18)	0.34 (0.27, 0.40)	±0.22 (0.11, 0.34)	0.87	0.85 (0.61, 0.95)
Cadence, steps/min	82.4 (15.9)	82.3 (15.7)	-0.08 (-0.62, 0.46)	±1.86 (0.92, 2.79)	0.99	0.99 (0.99, 0.99)
Gait Speed, m/s	0.45 (0.17)	0.68 (0.16)	0.23 (0.18, 0.28)	±0.18 (0.09, 0.27)	0.85	0.85 (0.60, 0.95)

Note: Standard deviation (SD); Intraclass correlation coefficient (ICC); confidence interval (CI); limits of agreement (LoA); Pearson correlation coefficient (r). A positive mean bias value indicates the smartphone application (OneStep) overestimated the variable compared to the motion capture system (Vicon). A negative mean bias value indicates the smartphone application underestimated the variable compared to the motion capture system.

[ƒ]<0.30 (negligible); 0.30-0.50 (low); 0.50-0.70 (moderate); 0.70-0.90 (high); >0.90 (very high)

[¥]<0.50 (poor reliability); 0.50-0.75 (moderate reliability); 0.75-0.90 (good reliability); >0.90 (excellent reliability)

Table S3. Spatiotemporal comparison between motion capture system and smartphone application for downhill walking without an assistive device.

Population/Variable	Vicon Mean (SD)	OneStep Mean (SD)	Mean Bias (95% CI)	LoA (95% CI)	r^{f}	ICC[¥] (95% CI)
**Older Adults**						
Double Limb Stance, %	30.4 (4.9)	30.5 (3.6)	0.15 (-1.47, 1.78)	±6.96 (4.14, 9.78)	0.71	0.68 (0.35, 0.85)
Left Single Limb Stance, %	34.6 (2.4)	33.8 (1.6)	-0.78 (-1.57, .00)	±3.36 (2.00, 4.73)	0.72	0.67 (0.34, 0.85)
Right Single Limb Stance, %	34.9 (2.7)	35.5 (2.8)	0.64 (-0.55, 1.83)	±5.10 (3.04, 7.17)	0.57	0.56 (0.18, 0.80)
Left Swing, %	34.9 (2.7)	35.5 (2.8)	0.63 (-0.57, 1.82)	±5.11 (3.04, 7.18)	0.56	0.56 (0.17, 0.80)
Right Swing, %	34.6 (2.4)	33.8 (1.6)	-0.77 (-1.56, 0.01)	±3.36 (1.99, 4.72)	0.72	0.67 (0.33, 0.85)
Left Step Length, m	0.48 (0.09)	0.55 (0.07)	0.06 (0.05, 0.08)	±0.08 (0.05, 0.11)	0.92	0.89 (0.76, 0.95)
Right Step Length, m	0.47 (0.09)	0.57 (0.09)	0.10 (0.08, 0.12)	±0.09 (0.05, 0.13)	0.88	0.88 (0.72, 0.95)
Stride Length, m	0.96 (0.18)	1.12 (0.16)	0.17 (0.13, 0.19)	±0.14 (0.08, 0.19)	0.92	0.92 (0.81, 0.96)
Cadence, steps/min	105.5 (11.5)	105.4 (11.5)	-0.13 (-0.57, 0.32)	±1.89 (1.12, 2.66)	0.99	0.99 (0.99, 0.99)
Gait Speed, m/s	0.86 (0.17)	0.98 (0.16)	0.13 (0.09, 0.16)	±0.15 (0.09, 0.21)	0.92	0.91 (0.78, 0.96)
**Parkinson's Disease**						
Double Limb Stance, %	38.5 (8.4)	35.6 (5.2)	-2.93 (-5.35, -0.51)	±8.73 (4.53, 12.92)	0.90	0.80 (0.51, 0.93)
Left Single Limb Stance, %	30.7	31.8	1.07	±6.26	0.69	0.58

	(4.2)	(2.3)	(-0.66, 2.80)	(3.29, 9.26)		(0.12, 0.83)
Right Single Limb Stance, %	30.6<br>(4.6)	32.5<br>(3.7)	1.86<br>(0.74, 2.98)	±4.05<br>(2.11, 6.00)	0.90	0.88<br>(0.69, 0.95)
Left Swing, %	30.6<br>(4.5)	32.5<br>(3.7)	1.84<br>(0.73, 2.96)	±4.04<br>(2.11, 5.98)	0.90	0.88<br>(0.69, 0.95)
Right Swing, %	30.7<br>(4.2)	31.8<br>(2.3)	1.08<br>(-0.65, 2.82)	±6.28<br>(3.34, 9.29)	0.69	0.58<br>(0.12, 0.83)
Left Step Length, m	0.35<br>(0.1)	0.42<br>(0.11)	0.07<br>(0.05, 0.09)	±0.07<br>(0.04, 0.10)	0.95	0.95<br>(0.86, 0.98)
Right Step Length, m	0.35<br>(0.11)	0.45<br>(0.11)	0.09<br>(0.07, 0.12)	±0.09<br>(0.05, 0.14)	0.91	0.91<br>(0.76, 0.96)
Stride Length, m	0.71<br>(0.21)	0.87<br>(0.22)	0.16<br>(0.13, 0.19)	±0.11<br>(0.06, 0.17)	0.97	0.96<br>(0.90, 0.98)
Cadence, steps/min	94.5<br>(7.8)	93.8<br>(7.4)	-0.69<br>(-1.71, 0.34)	±3.71<br>(1.93, 5.49)	0.97	0.97<br>(0.91, 0.99)
Gait Speed, m/s	0.56<br>(0.19)	0.68<br>(0.2)	0.12<br>(0.09, 0.15)	±0.10<br>(0.05, 0.14)	0.97	0.97<br>(0.91, 0.99)
**Cerebrovascular Accident**						
Double Limb Stance, %	40.8<br>(6.6)	37.3<br>(4.8)	-3.50<br>(-7.37, 0.36)	±13.39<br>(6.78, 20.09)	0.35	0.33<br>(-0.21, 0.72)
Left Single Limb Stance, %	30.4<br>(4.1)	31.9<br>(4.4)	1.50<br>(-0.98, 3.98)	±8.60<br>(4.40, 12.90)	0.49	0.49<br>(-0.03, 0.80)
Right Single Limb Stance, %	28.6<br>(5.6)	30.7<br>(5.6)	2.05<br>(0.03, 4.07)	±7.00<br>(3.55, 10.50)	0.80	0.80<br>(0.50, 0.93)
Left Swing, %	28.7<br>(5.6)	30.7<br>(5.7)	2.01<br>(-0.01, 4.04)	±7.02<br>(3.55, 10.53)	0.80	0.80<br>(0.50, 0.93)
Right Swing, %	30.4<br>(4.0)	31.9<br>(4.4)	1.54<br>(-0.94, 4.02)	±8.56<br>(4.26, 12.83)	0.49	0.49<br>(-0.03, 0.80)

Left Step Length, m	0.27 (0.09)	0.39 (0.06)	0.11 (0.06, 0.16)	±0.17 (0.09, 0.25)	0.48	0.46 (-0.06, 0.78)
Right Step Length, m	0.31 (0.10)	0.43 (0.05)	0.11 (0.06, 0.17)	±0.18 (0.09, 0.27)	0.47	0.41 (-0.12, 0.76)
Stride Length, m	0.59 (0.14)	0.82 (0.11)	0.22 (0.16, 0.29)	±0.23 (0.12, 0.35)	0.61	0.60 (0.13, 0.85)
Cadence, steps/min	85.6 (18.6)	86.1 (19.0)	0.50 (-0.52, 1.51)	±3.52 (1.76, 5.28)	0.99	0.99 (0.98, 0.99)
Gait Speed, m/s	0.44 (0.17)	0.59 (0.17)	0.15 (0.09, 0.20)	±0.19 (0.09, 0.29)	0.83	0.83 (0.57, 0.94)

Note: Standard deviation (SD); Intraclass correlation coefficient (ICC); confidence interval (CI); limits of agreement (LoA); Pearson correlation coefficient (r). A positive mean bias value indicates the smartphone application (OneStep) overestimated the variable compared to the motion capture system (Vicon). A negative mean bias value indicates the smartphone application underestimated the variable compared to the motion capture system.

[£]<0.30 (negligible); 0.30-0.50 (low); 0.50-0.70 (moderate); 0.70-0.90 (high); >0.90 (very high)

[¥]<0.50 (poor reliability); 0.50-0.75 (moderate reliability); 0.75-0.90 (good reliability); >0.90 (excellent reliability)

Table S4. Spatiotemporal comparison between motion capture system and smartphone application for downhill walking with an assistive device.

Population/Variable	Vicon Mean (SD)	OneStep Mean (SD)	Mean Bias (95% CI)	LoA (95% CI)	r^{f}	ICC[¥] (95% CI)
**Parkinson's Disease**						
Double Limb Stance, %	34.2 (6.1)	32.5 (4.2)	-1.69 (-4.09, 0.71)	±6.70 (2.54, 10.85)	0.85	0.79 (0.37, 0.94)
Left Single Limb Stance, %	32.6 (3.2)	33.4 (2.1)	0.73 (-1.55, 3.01)	±6.39 (2.43, 10.36)	0.35	0.32 (-0.34, 0.77)
Right Single Limb Stance, %	33.1 (4.2)	34.0 (3.2)	0.94 (-0.46, 2.34)	±3.93 (1.49, 6.37)	0.89	0.86 (0.54, 0.96)
Left Swing, %	33.1 (4.2)	34.0 (3.2)	0.97 (-0.43, 2.36)	±3.91 (1.49, 6.34)	0.89	0.86 (0.54, 0.96)
Right Swing, %	32.6 (3.2)	33.3 (2.1)	0.70 (-1.58, 2.98)	±6.39 (2.48, 10.35)	0.35	0.32 (-0.34, 0.77)
Left Step Length, m	0.41 (0.09)	0.48 (0.09)	0.07 (0.04, 0.09)	±0.07 (0.03, 0.12)	0.92	0.92 (0.72, 0.98)
Right Step Length, m	0.43 (0.08)	0.51 (0.09)	0.08 (0.05, 0.11)	±0.08 (0.03, 0.13)	0.91	0.90 (0.66, 0.97)
Stride Length, m	0.84 (0.17)	0.99 (0.18)	0.15 (0.12, 0.18)	±0.09 (0.04, 0.15)	0.96	0.96 (0.86, 0.99)
Cadence, steps/min	92.2 (5.5)	91.9 (6.2)	-0.36 (-1.25, 0.53)	±2.49 (0.93, 4.04)	0.98	0.97 (0.91, 0.99)
Gait Speed, m/s	0.66 (0.15)	0.76 (0.16)	0.09 (0.07, 0.13)	±0.09 (0.04, 0.16)	0.95	0.95 (0.83, 0.98)
**Cerebrovascular Accident**						
Double Limb Stance, %	40.0 (11.2)	37.9 (5.6)	-2.09 (-7.69, 3.50)	±19.39 (9.69, 29.09)	0.50	0.40 (-0.14, 0.75)
Left Single Limb Stance, %	31.3	30.5	-0.72	±9.95	0.42	0.39

	(5.2)	(3.6)	(-3.58, 2.15)	(4.98, 14.93)		(-0.14, 0.75)
Right Single Limb Stance, %	28.6<br>(8.1)	31.4<br>(4.6)	2.80<br>(-0.45, 6.06)	±11.20<br>(5.50, 16.76)	0.73	0.63<br>(0.18, 0.86)
Left Swing, %	28.6<br>(8.1)	31.4<br>(4.6)	2.79<br>(-0.47, 6.05)	±11.21<br>(5.51, 16.77)	0.73	0.63<br>(0.18, 0.86)
Right Swing, %	31.2<br>(5.2)	30.5<br>(3.6)	-0.70<br>(-3.55, 2.14)	±9.87<br>(4.94, 14.81)	0.42	0.39<br>(-0.14, 0.75)
Left Step Length, m	0.26<br>(0.11)	0.40<br>(0.06)	0.14<br>(0.08, 0.21)	±0.21<br>(0.11, 0.32)	0.36	0.32<br>(-0.22, 0.72)
Right Step Length, m	0.32<br>(0.1)	0.42<br>(0.08)	0.10<br>(0.05, 0.15)	±0.18<br>(0.09, 0.27)	0.56	0.55<br>(0.05, 0.83)
Stride Length, m	0.58<br>(0.17)	0.83<br>(0.13)	0.24<br>(0.16, 0.33)	±0.31<br>(0.15, 0.46)	0.52	0.51<br>(-0.00, 0.81)
Cadence, steps/min	82.5<br>(15.3)	82.8<br>(15.4)	0.32<br>(-0.25, 0.90)	±2.00<br>(1.00, 3.00)	0.99	0.99<br>(0.99, 0.99)
Gait Speed, m/s	0.42<br>(0.17)	0.57<br>(0.15)	0.14<br>(0.07, 0.21)	±0.24<br>(0.12, 0.35)	0.75	0.74<br>(0.38, 0.91)

Note: Standard deviation (SD); Intraclass correlation coefficient (ICC); confidence interval (CI); limits of agreement (LoA); Pearson correlation coefficient (r). A positive mean bias value indicates the smartphone application (OneStep) overestimated the variable compared to the motion capture system (Vicon). A negative mean bias value indicates the smartphone application underestimated the variable compared to the motion capture system.

[f]<0.30 (negligible); 0.30-0.50 (low); 0.50-0.70 (moderate); 0.70-0.90 (high); >0.90 (very high)

[¥]<0.50 (poor reliability); 0.50-0.75 (moderate reliability); 0.75-0.90 (good reliability); >0.90 (excellent reliability)

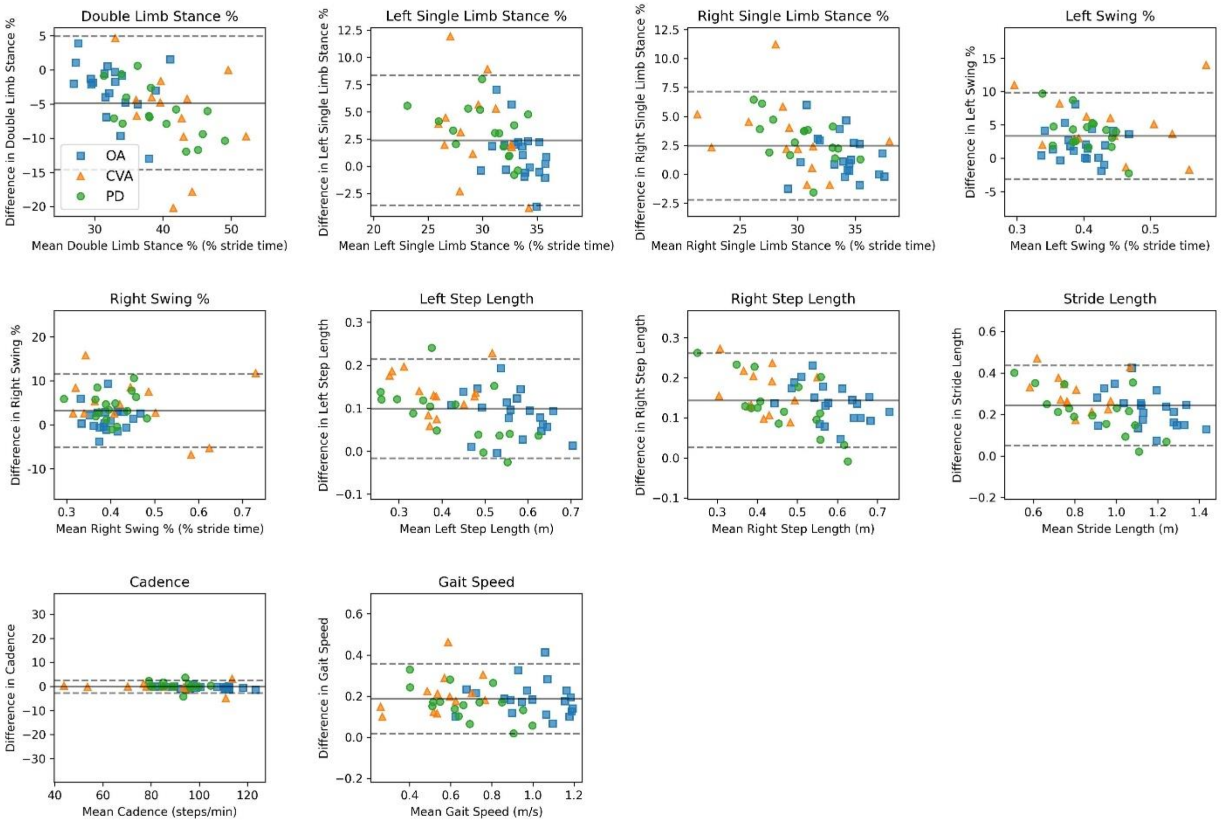

Figure S1. Bland-Altman plots comparing the smartphone application and motion capture system measurements in assessing spatiotemporal outcomes for incline walking without an assistive device across OA, PD, CVA. Mean bias is displayed as a solid line and 95% limits of agreement are displayed as dashed lines.

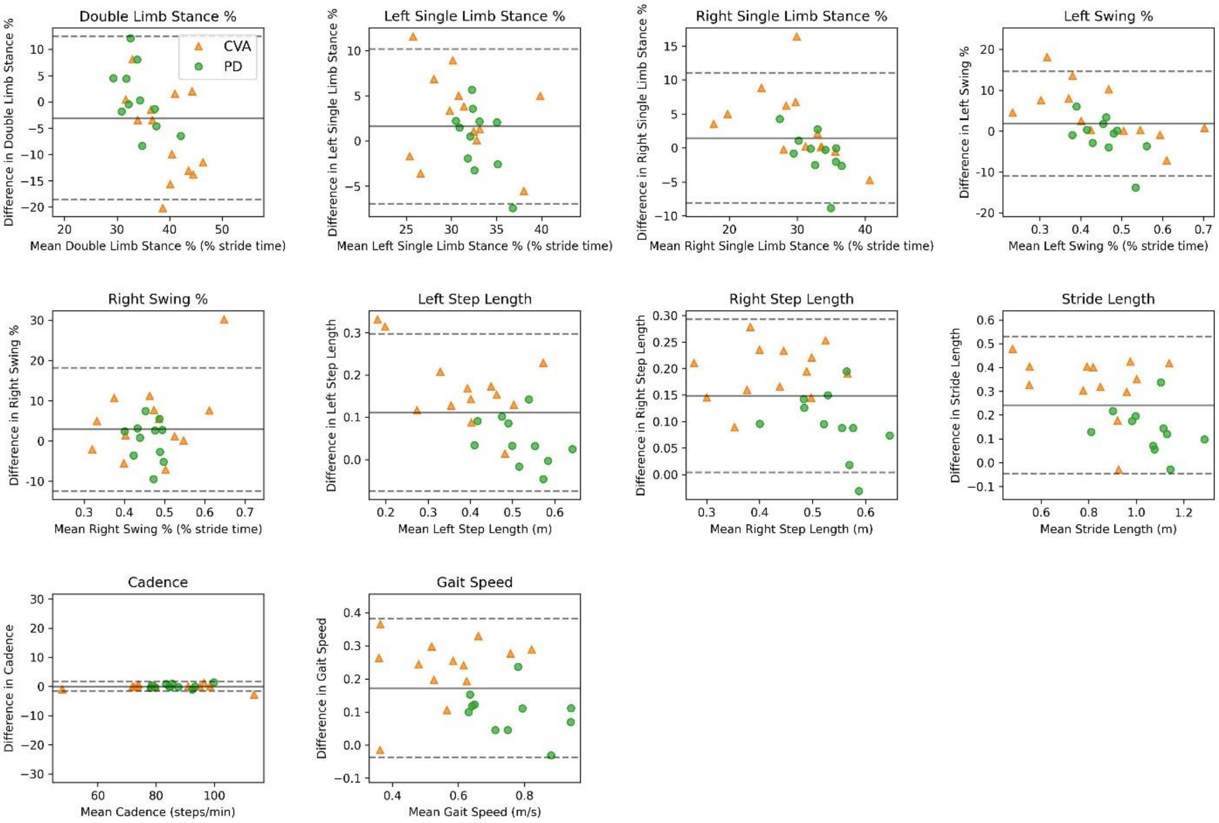

Figure S2. Bland-Altman plots comparing the smartphone application and motion capture system measurements in assessing spatiotemporal outcomes for incline walking with assistive device across PD and CVD. Mean bias is displayed as a solid line and 95% limits of agreement are displayed as dashed lines.

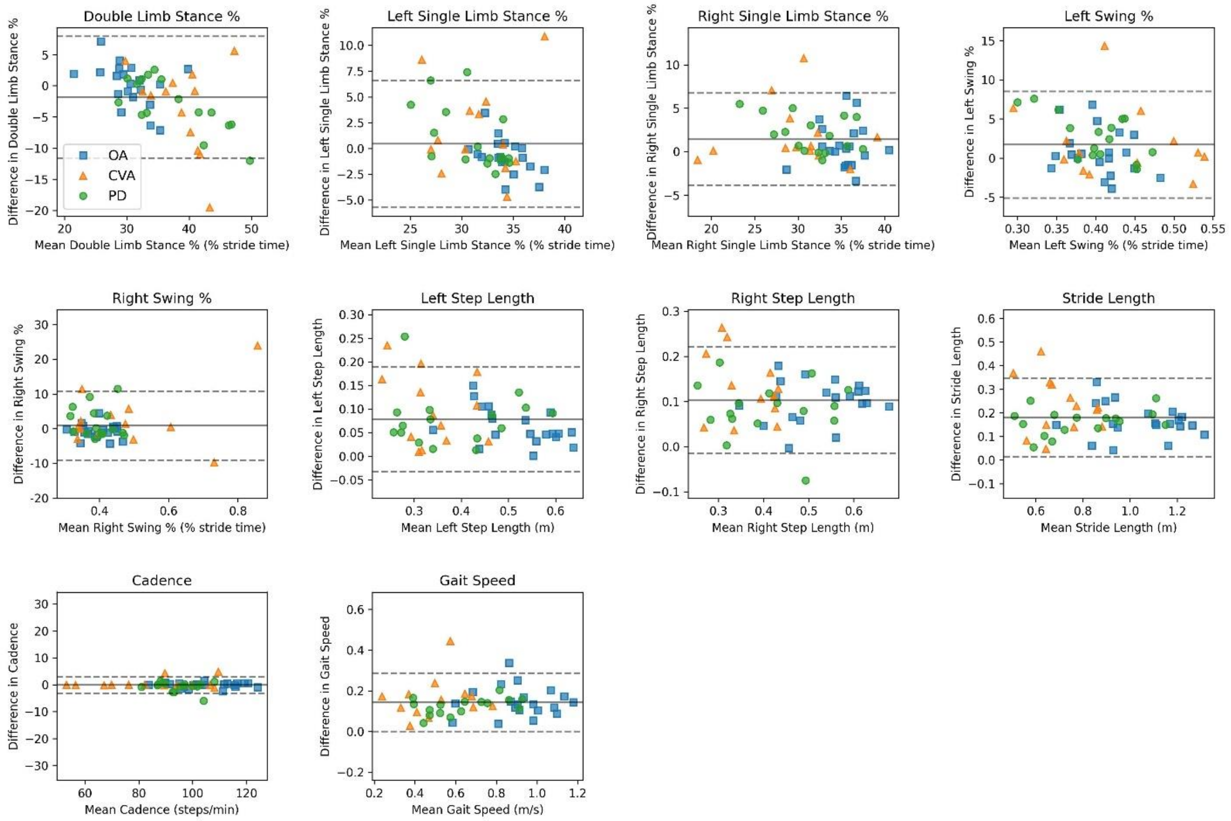

Figure S3. Bland-Altman plots comparing the smartphone application and motion capture system measurements in assessing spatiotemporal outcomes for decline walking without an assistive device across OA, PD, CVA. Mean bias is displayed as a solid line and 95% limits of agreement are displayed as dashed lines.

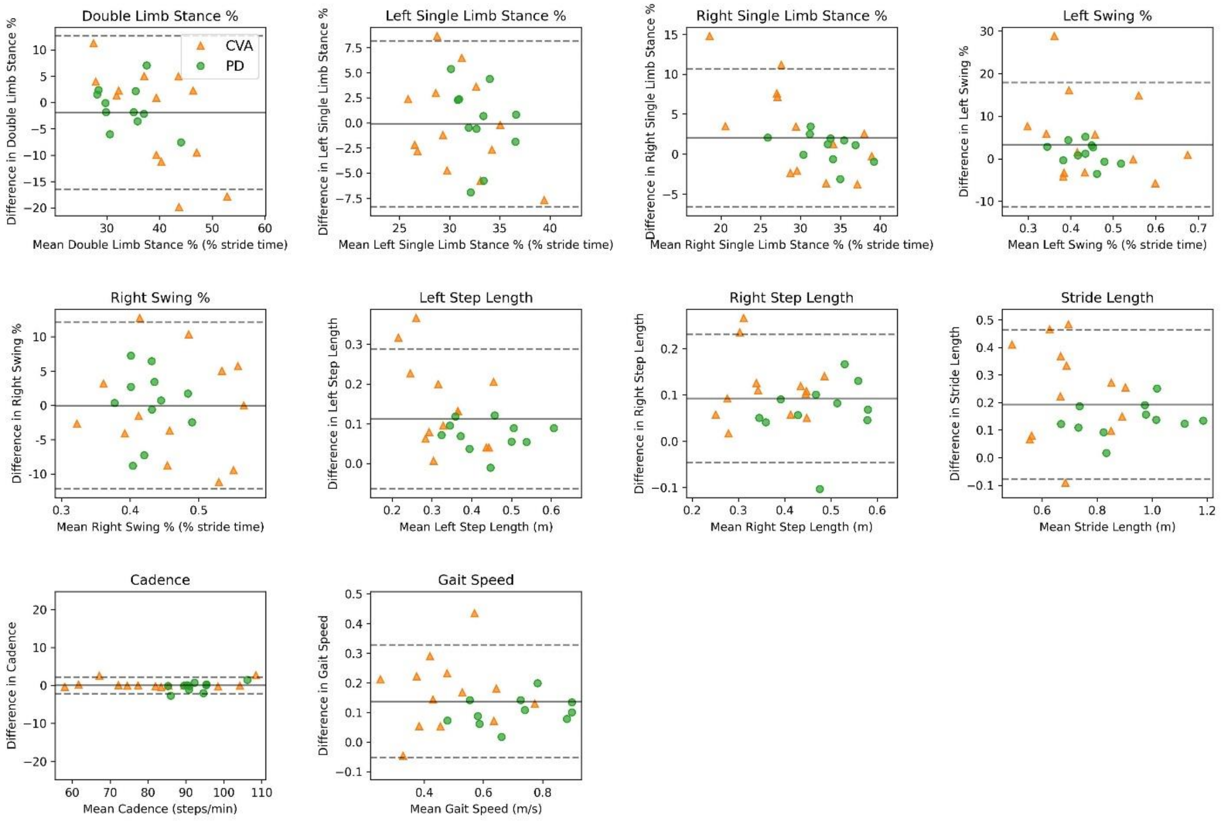

Figure S4. Bland-Altman plots comparing the smartphone application and motion capture system measurements in assessing spatiotemporal outcomes for decline walking with assistive device across PD and CVD. Mean bias is displayed as a solid line and 95% limits of agreement are displayed as dashed lines.
